# Vaccination Coverage Among Adolescents Aged 13–17 Years — National Immunization Survey–Teen, United States, 2022

**DOI:** 10.15585/mmwr.mm7234a3

**Published:** 2023-08-25

**Authors:** Cassandra Pingali, David Yankey, Laurie D. Elam-Evans, Lauri E. Markowitz, Madeleine R. Valier, Benjamin Fredua, Samuel J. Crowe, Carla L. DeSisto, Shannon Stokley, James A. Singleton

**Affiliations:** ^1^Immunization Services Division, National Center for Immunization and Respiratory Diseases, CDC; ^2^Division of Viral Diseases, National Center for Immunization and Respiratory Diseases, CDC; ^3^Oak Ridge Institute for Science and Education, Oak Ridge, Tennessee; ^4^Leidos Health, Inc., Atlanta, Georgia; ^5^Division of Bacterial Diseases, National Center for Immunization and Respiratory Diseases, CDC.

**Summary**

**What is already known about this topic?**

Tetanus, diphtheria, and acellular pertussis vaccine, meningococcal conjugate vaccine, and human papillomavirus (HPV) vaccine are routinely recommended for children at age 11–12 years.

**What is added by this report?**

Analyses of recent trends in routine vaccination coverage show declines in coverage by age 13 and 14 years among adolescents born in 2008. Among adolescents aged 13–17 years, routine vaccination coverage in 2022 was similar to coverage in 2021. Coverage with ≥1 HPV vaccine dose declined among adolescents insured by Medicaid.

**What are the implications for public health?**

Providers should review adolescent immunization histories, particularly those of adolescents born in 2008 and those eligible for the Vaccines for Children program, to ensure that adolescents are up to date with all recommended vaccinations.

## Abstract

Three vaccines are routinely recommended for adolescents to prevent pertussis, meningococcal disease, and cancers caused by human papillomavirus (HPV). CDC analyzed data from the 2022 National Immunization Survey–Teen for 16,043 adolescents aged 13–17 years to assess vaccination coverage. Birth cohort analyses were conducted to assess trends in vaccination coverage by age 13 years (i.e., before the 13th birthday) and by age 14 years (i.e., before the 14th birthday) among adolescents who were due for routine vaccination before and during the COVID-19 pandemic. Cross-sectional analysis was used to assess coverage estimates among adolescents aged 13–17 years. In 2022, vaccination coverage by age 14 years among adolescents born in 2008 continued to lag that of earlier birth cohorts and varied by sociodemographic factors and access to health care compared with coverage among earlier birth cohorts. Vaccination coverage by age 13 years among adolescents born in 2009 was similar to coverage estimates obtained before the COVID-19 pandemic. Among all adolescents aged 13–17 years, 2022 vaccination coverage levels did not differ from 2021 levels; however, initiation of the HPV vaccination series decreased among those who were insured by Medicaid. Coverage with ≥1 dose of tetanus, diphtheria, and acellular pertussis vaccine and ≥1 dose meningococcal conjugate vaccine was high and stable (around 90%). Providers should review adolescent vaccination records, especially among those born in 2008 and those in populations eligible for the Vaccines for Children program, to ensure adolescents are up to date with all recommended vaccines.

## Introduction

In the United States, the Advisory Committee on Immunization Practices (ACIP) recommends that children aged 11–12 years receive tetanus, diphtheria, and acellular pertussis vaccine (Tdap), meningococcal conjugate vaccine (MenACWY), and human papillomavirus (HPV) vaccine (HPV vaccine can be started at age 9 years). A booster dose of MenACWY is recommended at age 16 years, and using shared clinical decision-making, adolescents and young adults aged 16–23 years may also receive serogroup B meningococcal vaccine (MenB). ACIP also recommends that adolescents stay up to date with COVID-19 vaccines,* acquire any missed childhood vaccines (catch-up vaccination), and receive an annual influenza vaccine^†^ (*1*). Results from 2021 National Immunization Survey–Teen (NIS-Teen) revealed declines in MenACWY^§^ and Tdap^¶^ coverage among adolescents born in 2008; these persons were due for their routine adolescent vaccines in 2020, during the height of the COVID-19 pandemic (*2*). Ongoing assessment of adolescent vaccination coverage can help guide progress in implementation of ACIP recommendations and identify populations and areas with low coverage.

## Methods

NIS-Teen is a random-digit–dialed telephone survey** conducted among households that include adolescents aged 13–17 years in the 50 states, the District of Columbia, selected local areas, and some U.S. territories.^††^ Parents and guardians are interviewed to obtain adolescent, maternal, and household information and are asked to provide consent for their adolescent’s vaccine providers to be contacted. Immunization history questionnaires are mailed to all vaccine providers identified by the parent or guardian to obtain the adolescent’s complete vaccination record. The 2022 NIS-Teen vaccination coverage estimates were based on provider-reported vaccination histories from 16,043 adolescents aged 13–17 years^§§^ who were born during January 2004–January 2010^¶¶^ and included any vaccines received before the household interview date. Recent trends in vaccination coverage were assessed by comparing vaccination coverage by age among the 2008 and 2009 birth cohorts (i.e., those who reached their 12th and 11th birthdays, respectively, in 2020) to vaccination coverage in earlier birth cohorts (i.e., adolescents born in 2006 and 2007) whose routine vaccinations were not affected by the pandemic. Cross-sectional analysis was used to estimate vaccination coverage among adolescents aged 13–17 years. The household response rate*** was 23.0%, and 38.8% of adolescents with completed interviews had adequate provider data.^†††^ To better understand recent trends in vaccination coverage, estimates by age and birth year (2006–2009) were obtained; Kaplan-Meier techniques were used to account for censoring of vaccination status at age ≥14 years. Z-tests were used to compare differences in vaccination coverage by survey year, birth year, and among sociodemographic groups; differences with p-values <0.05 were considered statistically significant. Data were weighted^§§§^ and analyses were conducted using SAS-callable SUDAAN (version 11; RTI International). This activity was reviewed by CDC and was conducted consistent with applicable federal law and CDC policy.^¶¶¶^

## Results

### Vaccination Coverage Among Adolescents Aged 13–17 Years

In 2022, coverage with all routine, catch-up,**** and other^††††^ vaccinations recommended for adolescents was similar to coverage in 2021 (Table 1) ( Supplementary Figure 1, https://stacks.cdc.gov/view/cdc/131939). In 2022, 89.9% of adolescents aged 13–17 years had received ≥1 Tdap dose, 88.6% had received ≥1 MenACWY dose, 76.0% had received ≥1 HPV^§§§§^ vaccine dose, and 62.6% were up to date with HPV vaccination (HPV UTD).^¶¶¶¶^ During 2015–2021, among adolescents aged 13–17 years, coverage with ≥1 HPV vaccine dose was higher among those insured by Medicaid than among those with private insurance (Supplementary Figure 2, https://stacks.cdc.gov/view/cdc/131940); however, in 2022, coverage with ≥1 HPV vaccine dose among Medicaid beneficiaries declined by 3.3 percentage points compared with coverage in 2021, whereas ≥1-dose HPV coverage among those with private insurance was stable, resulting in similar coverage between the two groups in 2022. Coverage with ≥1 HPV vaccine dose remains lowest among uninsured adolescents. Coverage with all routine vaccines varied widely by jurisdiction (Supplementary Table, https://stacks.cdc.gov/view/cdc/132006). Coverage with ≥1 Tdap dose ranged from 82.7% in California to 97.3% in Iowa, and ≥1-dose MenACWY coverage ranged from 55.5% in Mississippi to 97.9% in Iowa. Coverage with ≥1 HPV vaccine dose ranged from 61.0% in Mississippi to 94.6% in Rhode Island, and the percentage of adolescents UTD with HPV vaccine ranged from 38.5% in Mississippi to 85.2% in Rhode Island.

**TABLE 1:** Estimated vaccination coverage with selected vaccines and doses among adolescents aged 13–17 years,* by age at interview — National Immunization Survey–Teen, United States, 2022

Vaccine/Population group	Age at 2022 interview, yrs, % (95% CI)^†^					Total, % (95% CI)^†^	
	13 (n = 3,198)	14 (n = 3,399)	15 (n = 3,219)	16 (n = 3,208)	17 (n = 3,019)	2022 (N = 16,043)	2021 (N = 18,002)
**Tdap ≥1 dose^§^**	85.1 (81.7–88.0)	90.7 (88.8–92.3)^¶^	91.5 (89.6–93.1)^¶^	91.1 (88.9–93.0)^¶^	91.4 (89.2–93.2)^¶^	**89.9 (88.9–90.9)**	**89.6 (88.6–90.5)**
MenACWY**							
≥1 dose	84.5 (81.3–87.2)	89.2 (87.1–91.0)^¶^	89.0 (86.7–91.0)^¶^	89.8 (87.4–91.8)^¶^	90.7 (88.7–92.3)^¶^	**88.6 (87.6–89.6)**	**89.0 (87.9–90.0)**
≥2 doses^††^	NA	NA	NA	NA	60.8 (57.5–63.9)	**60.8 (57.5–63.9)**	**60.0 (56.6–63.3)**
**MenB^§§^**							
≥1 dose	NA	NA	NA	NA	29.4 (26.5–32.4)^¶^	**29.4 (26.5–32.4)**	**31.4 (28.2–34.8)**
≥2 doses	NA	NA	NA	NA	11.9 (10.0–14.1)	**11.9 (10.0–14.1)**	**NA**
**HPV^¶¶^ vaccine**							
**All adolescents**							
≥1 dose	68.9 (65.4–72.2)	75.8 (73.0–78.4)^¶^	78.5 (75.7–81.1)^¶^	79.6 (76.8–82.2)^¶^	77.4 (74.5–80.0)^¶^	**76.0 (74.7–77.3)**	**76.9 (75.6–78.2)**
HPV vaccine UTD***	50.0 (46.4–53.5)	60.3 (57.1–63.4)^¶^	65.8 (62.7–68.8)^¶^	68.8 (65.8–71.7)^¶^	68.3 (65.3–71.2)^¶^	**62.6 (61.1–64.0)**	**61.7 (60.2–63.2)**
**Females**							
≥1 dose	72.8 (67.7–77.3)	76.5 (72.4–80.2)	79.5 (75.3–83.2)^¶^	81.3 (76.8–85.1)^¶^	79.0 (75.0–82.5)^¶^	**77.8 (75.8–79.6)**	**78.5 (76.6–80.4)**
HPV UTD	52.3 (47.1–57.4)	61.7 (57.3–65.9)^¶^	68.5 (63.9–72.8)^¶^	70.8 (66.2–75.0)^¶^	70.9 (66.7–74.8)^¶^	**64.6 (62.5–66.6)**	**63.8 (61.5–65.9)**
**Males**							
≥1 dose	65.0 (60.0–69.7)	75.1 (71.2–78.7)^¶^	77.5 (73.7–80.9)^¶^	78.1 (74.3–81.4)^¶^	75.9 (71.8–79.5)^¶^	**74.4 (72.5–76.1)**	**75.4 (73.5–77.2)**
HPV UTD	47.6 (42.8–52.4)	58.8 (54.3–63.2)^¶^	63.3 (59.0–67.4)^¶^	67.0 (62.9–70.8)^¶^	66.0 (61.7–70.0)^¶^	**60.6 (58.6–62.6)**	**59.8 (57.6–61.8)**
**MMR ≥2 doses**	90.5 (87.6–92.8)	92.6 (90.4–94.4)	91.0 (88.7–92.8)	92.0 (90.0–93.6)	89.9 (87.3–92.0)	**91.2 (90.2–92.1)**	**92.2 (91.2–93.2)**
**Hepatitis A vaccine ≥2 doses^†††^**	84.8 (81.5–87.5)	86.6 (83.9–88.9)	86.7 (84.2–88.8)	84.6 (81.9–87.0)	82.3 (79.7–84.7)	**85.0 (83.8–86.1)**	**85.0 (83.8–86.1)**
**Hepatitis B vaccine ≥3 doses**	90.5 (87.6–92.8)	92.6 (90.6–94.2)	91.0 (88.8–92.8)	91.2 (88.9–93.1)	90.7 (88.3–92.6)	**91.2 (90.2–92.1)**	**92.3 (91.3–93.1)**
**Varicella**							
History of varicella^§§§^	4.6 (3.4–6.3)	6.4 (5.0–8.1)	7.4 (5.8–9.3)^¶^	7.4 (5.9–9.2)^¶^	9.3 (7.5–11.4)^¶^	**7.0 (6.3–7.8)**	**7.3 (6.5–8.2)**
No history of varicella disease							
≥1 dose vaccine	93.5 (90.9–95.5)	95.2 (93.5–96.5)	93.4 (91.1–95.1)	94.4 (92.8–95.6)	93.8 (91.4–95.5)	**94.1 (93.2–94.8)**	**94.9 (94.0–95.7)**
≥2 doses vaccine	89.4 (86.2–91.9)	91.9 (89.5–93.8)	91.3 (89.1–93.2)	91.1 (89.0–92.9)	90.4 (87.9–92.4)	**90.8 (89.8–91.8)**	**91.5 (90.5–92.5)**
History of varicella or receipt of ≥2 varicella vaccine doses	89.9 (86.9–92.3)	92.4 (90.1–94.2)	92.0 (89.9–93.7)	91.8 (89.8–93.4)	91.3 (89.0–93.1)	**91.5 (90.5–92.4)**	**92.2 (91.2–93.1)**

**Abbreviations:** HPV = human papillomavirus; MenACWY = quadrivalent meningococcal conjugate vaccine; MenB = serogroup B meningococcal vaccine; MMR = measles, mumps, and rubella vaccine; NA = not applicable; NIS = National Immunization Survey; Tdap = tetanus, diphtheria, and acellular pertussis vaccine; UTD = up to date.

* Adolescents in the 2022 NIS–Teen were born during January 7, 2004–January 10, 2010.

^†^ Estimates with 95% CIs widths >20 might not be reliable.

^§^ Includes percentages receiving Tdap vaccine at age ≥10 years.

^¶^ Statistically significant difference (p<0.05) in estimated vaccination coverage by age; referent group was adolescents aged 13 years.

** Includes percentages receiving MenACWY or an unknown type of meningococcal vaccine.

^††^ ≥2 doses of MenACWY or unknown type of meningococcal vaccine among adolescents aged 17 years at interview and does not include adolescents who received 1 dose of MenACWY vaccine at age ≥16 years.

^§§^ Calculated only among adolescents who were aged 17 years at time of interview with vaccine administered based on individual clinical decision.

^¶¶^ HPV vaccine, nine-valent (9vHPV), quadrivalent (4vHPV), or bivalent (2vHPV). For ≥1 dose and HPV UTD measures, percentages are reported among females and males combined (16,043) and for females only (7,623) and males only (8,420).

*** HPV vaccine UTD includes those with ≥3 doses, and those with 2 doses when the first HPV vaccine dose was initiated at age <15 years and there were ≥5 months minus 4 days between the first and second dose (https://www.cdc.gov/vaccines/programs/iis/cdsi.html). This update to the HPV recommendation occurred in December 2016.

^†††^ In July 2020, ACIP revised recommendations for Hepatitis A vaccination to include catch-up vaccination for persons aged 2–18 years who have not previously received Hepatitis A vaccine at any age. https://pubmed.ncbi.nlm.nih.gov/32614811/

^§§§^ By parent or guardian report or provider records.

### Trends in Vaccination Coverage by Age 13 and by Age 14 Years

Vaccination coverage by age 13 years among adolescents born in 2009 was similar to that attained by those born in 2006 and 2007 for all vaccinations recommended for adolescents***** (Figure). By age 13 years, coverage with ≥1 Tdap was 3.2 percentage points lower in the 2008 birth cohort than in the 2007 birth cohort, and coverage with ≥1 MenACWY dose was 3.0 percentage points lower (Table 2). By age 14 years, coverage rates with ≥1 Tdap dose, ≥1 HPV dose, and HPV UTD status were 3.8, 3.8, and 5.7 percentage points lower in the 2008 birth cohort than in the 2007 birth cohort, respectively.

**FIGURE:**
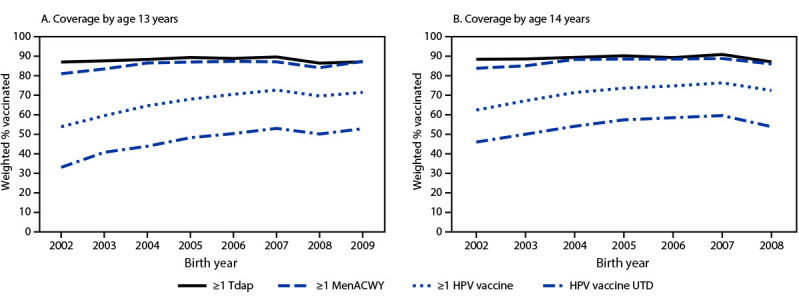
**Estimated coverage with ≥1 dose of tetanus, diphtheria, and acellular pertussis vaccine, ≥1 dose of quadrivalent meningococcal conjugate vaccine, and ≥1 dose of human papillomavirus vaccine, and percentage of adolescents up to date with human papillomavirus vaccination, among adolescents born during 2002–2009** * **, by age 13 years** ^†^ **(A) and 14 years** ^§^ **(B) — National Immunization Survey-Teen, United States, 2015–2022** **Abbreviations:** HPV = human papillomavirus; MenACWY = quadrivalent meningococcal conjugate vaccine; Tdap = tetanus toxoid, reduced diphtheria toxoid, and acellular pertussis vaccine; UTD = up to date. * The 2008 and 2009 birth cohorts reached their 12th and 11th birthdays, respectively, in 2020 during the COVID-19 pandemic. ^^†^^ Includes vaccinations received before the 13th birthday. ^^§^^Includes vaccinations received before the 14th birthday.

**TABLE 2:** Coverage with ≥1 dose of tetanus, diphtheria, and acellular pertussis vaccine, ≥1 dose of quadrivalent meningococcal conjugate vaccine, ≥1 dose of human papillomavirus vaccine, and percentage of adolescents up to date with human papillomavirus vaccination, among adolescents born during 2006–2009,* by age 13 years and 14 years,^†^ metropolitan statistical area status,^§^ poverty status,^¶^ race and ethnicity,** and health insurance status^††^ — National Immunization Survey-Teen, United States, 2020–2022

Age group/ Characteristic	Vaccination coverage,% (95% CI)§§															
	≥1 Tdap				≥1 MenACWY				≥1 HPV				HPV Vaccine UTD			
	Birth year				Birth year				Birth year				Birth year			
	2006	2007	2008	2009	2006	2007	2008	2009	2006	2007	2008	2009	2006	2007	2008	2009
**By age 13 yrs**																
All adolescents	88.8 (87.7–89.9)	89.6 (88.5–90.7)	86.4 (84.1–88.5)^¶¶^	87.1 (83.0–90.7)	87.3 (86.0–88.5)	87.1 (85.5–88.5)	84.1 (81.5–86.4)^¶¶^	87.3 (84.1–90.2)	70.4 (68.8–72.0)	72.6 (70.8–74.5)	69.5 (66.8–72.1)	71.4 (67.1–75.6)	50.2 (48.5–51.9)	52.9 (50.8–55.0)	50.0 (47.2–52.8)	52.7 (48.0–57.6)
**MSA**																
MSA, principal city	88.4 (86.4–90.2)	90.0 (88.3–91.5)	86.6 (83.2–89.7)	86.3 (78.5–92.3)	86.7 (84.4–88.9)	89.0 (87.2–90.7)	81.8 (77.3–85.8)^¶¶^	87.8 (82.2–92.3)	73.3 (70.7–75.9)	77.9 (75.4–80.3)	69.4 (64.8–73.8)^¶¶^	74.8 (67.4–81.7)	52.4 (49.6–55.1)	56.8 (53.6–60.0)	49.2 (44.6–54.0)^¶¶^	55.1 (47.3–63.3)
MSA, nonprincipal city	90.0 (88.4–91.4)	89.3 (87.4–91.0)	85.5 (81.9–88.7)	87.6 (82.4–91.8)	89.0 (87.4–90.5)	85.7 (82.9–88.2)	85.6 (82.0–88.8)	87.3 (82.9–91.1)	69.3 (66.9–71.6)	68.8 (65.7–71.8)	69.9 (66.1–73.7)	69.3 (63.3–75.0)	49.6 (47.1–52.1)	50.8 (47.6–53.9)	50.7 (46.9–54.7)	52.5 (46.2–59.2)
Non–MSA	85.6 (82.4–88.4)	89.9 (87.3–92.1)	89.5 (85.4–92.9)	88.6 (79.2–95.0)	81.8 (78.8–84.6)	85.4 (82.3–88.1)	86.2 (82.0–89.9)	84.9 (75.3–92.3)	64.3 (60.4–68.1)	69.3 (65.3–73.2)	67.7 (61.1–74.2)	67.0 (56.3–77.4)	44.6 (40.4–49.1)	47.1 (42.5–52.0)	49.5 (43.3–56.2)	43.3 (32.0–56.6)
**Poverty status**																
At or above federal poverty level	89.1 (87.8–90.3)	89.4 (88.2–90.6)	86.6 (84.1–88.8)^¶¶^	88.5 (84.6–91.8)	87.5 (86.0–88.9)	87.3 (85.6–89.0)	85.2 (82.6–87.5)	87.6 (83.8–90.8)	68.8 (67.0–70.5)	71.2 (69.1–73.3)	68.5 (65.6–71.4)	70.2 (65.2–75.0)	49.4 (47.6–51.3)	52.4 (50.1–54.7)	49.5 (46.6–52.5)	51.1 (45.9–56.4)
Below federal poverty level	89.1 (86.9–91.1)	90.6 (87.2–93.3)	83.0 (75.9–88.9)^¶¶^	89.5 (82.6–94.5)	86.8 (84.2–89.3)	86.0 (82.0–89.5)	78.1 (69.6–85.6)	89.4 (82.6–94.4) ***	79.0 (75.5–82.2)	79.1 (74.6–83.3)	74.3 (66.8–81.2)	77.7 (68.7–85.6)	54.3 (50.1–58.7)	55.0 (49.4–60.7)	52.6 (44.6–61.1)	57.8 (46.6–69.4)
**Race and ethnicity**																
AI/AN, NH	79.4 (57.3–94.7)	92.5 (85.4–96.9)	91.1 (78.0–97.9)	NA	79.1 (57.1–94.4)	82.4 (67.9–93.0)	83.6 (68.8–94.0)	NA	63.6 (45.8–81.2)	72.5 (52.9–89.1)	69.1 (51.4–85.1)	NA	49.2 (34.3–66.5)	54.4 (35.9–75.1)	51.3 (33.9–71.3)	31.8 (14.2–61.6)
Asian, NH	87.3 (81.7–91.8)	84.0 (76.1–90.4)	87.5 (77.6–94.4)	75.4 (50.2–94.0)	91.4 (87.1–94.8)	88.1 (81.9–93.0)	94.4 (89.5–97.5)	NA	76.8 (70.6–82.4)	76.7 (68.7–83.9)	64.6 (52.2–76.8)	57.6 (34.4–82.6)	58.5 (50.9–66.3)	60.7 (50.6–71.0)	50.7 (39.5–63.1)	49.2 (28.6–74.3)
Black or African American, NH	88.5 (85.6–91.1)	90.3 (87.2–92.9)	84.6 (78.8–89.5)	89.0 (80.8–94.7)	87.8 (84.6–90.6)	86.1 (81.6–89.9)	81.8 (74.7–87.9)	82.6 (72.8–90.5)	76.6 (72.9–80.2)	79.4 (74.3–84.0)	72.6 (65.4–79.4)	70.7 (60.1–80.6)	53.9 (49.4–58.6)	57.3 (51.7–63.1)	54.4 (47.3–61.8)	54.9 (43.1–67.5)
Hispanic or Latino	87.4 (84.1–90.3)	89.2 (86.3–91.7)	84.4 (77.7–90.0)	82.7 (71.3–91.5)	86.8 (83.2–90.1)	86.6 (82.2–90.3)	81.0 (73.4–87.5)	87.8 (79.5–93.8)	72.8 (68.6–76.8)	74.4 (69.6–79.0)	74.5 (67.6–80.9)	78.6 (69.7–86.4)	54.8 (50.5–59.2)	54.8 (49.8–60.1)	52.9 (45.7–60.4)	57.8 (47.2–68.8)
White, NH	89.9 (88.7–91.1)	90.2 (88.8–91.5)	87.7 (85.3–90.0)	91.3 (88.1–93.9)	87.0 (85.5–88.4)	87.2 (85.5–88.7)	85.4 (82.7–87.8)	89.4 (86.1–92.2)	66.8 (64.9–68.6)	69.2 (67.0–71.5)	66.8 (63.6–70.1)	67.0 (61.1–72.7)	46.0 (44.1–47.9)	50.0 (47.6–52.5)	46.7 (43.6–49.8)	47.5 (41.8–53.6)
**Health insurance status**																
Private insurance only	89.6 (88.1–91.0)	91.0 (89.7–92.3)	87.9 (85.3–90.2)^¶¶^	89.0 (83.8–93.1)	88.6 (87.2–90.0)	88.3 (86.1–90.3)	86.8 (84.0–89.4)	92.3 (89.1–94.8) ^¶¶,^***	68.9 (66.9–70.9)	71.2 (68.5–73.8)	69.8 (66.4–73.2)	73.9 (68.3–79.2)	50.5 (48.4–52.6)	52.7 (49.8–55.5)	50.4 (47.0–53.9)	55.2 (49.1–61.5)
Any Medicaid insurance	88.5 (86.3–90.4)	88.8 (86.6–90.9)	84.5 (80.5–88.2)	86.5 (78.6–92.6)	87.1 (84.6–89.4)	86.1 (83.6–88.4)	82.0 (77.4–86.1)	84.0 (78.2–89.0)	74.2 (71.2–77.1)	75.6 (72.6–78.5)	71.8 (67.3–76.2)	70.6 (63.2–77.8)	52.4 (49.3–55.6)	55.3 (51.8–58.9)	51.9 (47.0–56.9)	52.2 (44.0–60.9)
Other insurance	88.6 (85.5–91.3)	88.8 (85.0–92.1)	94.2 (91.4–96.3)^¶¶^	79.0 (66.4–89.4)	85.8 (81.9–89.3)	88.7 (85.0–91.8)	86.7 (76.6–93.9)	75.5 (64.1–85.5)	70.2 (65.4–74.9)	74.0 (68.7–79.0)	66.1 (55.9–76.0)	63.8 (51.7–75.8)	45.0 (39.6–50.8)	50.2 (43.8–56.9)	48.5 (38.2–59.8)	45.6 (33.5–59.7)
Uninsured	80.2 (71.9–87.3)	79.2 (69.1–87.8)	71.1 (49.3–89.7)	82.1 (55.4–97.4)	69.7 (59.7–79.1)	75.3 (63.3–85.8)	63.2 (42.7–83.3)	74.5 (43.4–96.3)	NA	58.0 (46.5–70.0)	46.5 (29.9–66.7)	59.9 (34.1–86.4)	NA	34.9 (25.0–47.3)	26.9 (15.1–45.0)	39.3 (18.4–70.5)
**By age 14 yrs^†††^**																
All adolescents	89.3 (88.2–90.4)	90.9 (89.7–91.9)	87.1 (84.9–89.2) ^§§§^	NA	88.5 (87.3–89.7)	88.8 (87.2–90.2)	86.0 (83.2–88.6)	NA	74.8 (73.1–76.4)	76.3 (74.4–78.2)	72.5 (69.5–75.5) ^§§§^	NA	58.5 (56.7–60.3)	59.6 (57.4–61.9)	53.9 (50.9–56.9) ^§§§,¶¶¶^	NA
**MSA**																
MSA, principal city	88.9 (86.9–90.7)	91.2 (89.5–92.7)	87.3 (83.8–90.4) ^§§§^	NA	88.5 (86.3–90.5)	90.8 (89.1–92.4)	83.3 (78.7–87.3) ^§§§,¶¶¶^	NA	78.4 (75.8–80.8)	80.3 (77.8–82.7)	71.9 (67.0–76.5) ^§§§,¶¶¶^	NA	60.9 (58.0–63.8)	63.8 (60.4–67.2)	52.1 (47.2–57.2) ^§§§,¶¶¶^	NA
MSA, Nonprincipal city	90.2 (88.6–91.7)	90.7 (88.8–92.4)	86.4 (82.7–89.6) ^§§§^	NA	89.6 (87.9–91.1)	87.4 (84.6–90.0)	88.3 (84.0–91.8)	NA	73.3 (70.9–75.7)	73.7 (70.4–76.8)	74.0 (69.6–78.3)	NA	57.7 (55.1–60.3)	56.9 (53.6–60.3)	55.3 (51.2–59.6)	NA
Non–MSA	87.2 (84.1–89.9)	90.2 (87.6–92.5)	90.1 (86.1–93.4)	NA	84.0 (81.1–86.7)	86.4 (83.4–89.1)	87.0 (82.8–90.6)	NA	66.9 (63.1–70.8)	72.2 (68.2–76.1)	68.2 (61.6–74.6)	NA	52.2 (47.9–56.7)	55.7 (50.0–61.5)	54.9 (48.3–61.7)	NA
**Poverty status**																
At or above poverty level	89.6 (88.3–90.8)	90.6 (89.3–91.7)	86.7 (84.2–89.0) ^§§§,¶¶¶^	NA	88.9 (87.5–90.2)	88.9 (87.2–90.5)	86.8 (83.8–89.4)	NA	73.9 (72.1–75.6)	75.2 (73.0–77.3)	71.9 (68.5–75.2)	NA	57.5 (55.6–59.4)	58.9 (56.5–61.4)	53.0 (50.0–56.1) ^§§§,¶¶¶^	NA
Below poverty level	89.6 (87.4–91.5)	92.4 (88.9–95.2)	86.4 (79.4–91.9)	NA	87.9 (85.2–90.2)	88.6 (84.5–92.1)	80.1 (71.5–87.5)	NA	80.8 (77.3–84.0)	82.0 (77.4–86.2)	75.3 (67.7–82.3)	NA	63.8 (59.4–68.2)	61.2 (55.4–67.0)	55.2 (46.8–64.0)	NA
**Race and ethnicity**																
Asian, NH	87.8 (82.2–92.3)	84.8 (76.9–91.2)	90.3 (79.6–96.7)	NA	92.3 (88.0–95.6)	88.9 (82.7–93.7)	94.6 (89.7–97.6)	NA	80.2 (74.1–85.6)	83.8 (76.5–89.8)	72.6 (56.4–86.7)	NA	67.1 (59.7–74.4)	67.4 (57.8–76.8)	58.1 (44.6–72.2)	NA
AI/AN, NH	NA	94.2 (87.0–98.1)	NA	NA	79.2 (57.2–94.6)	85.2 (70.1–95.1)	NA	NA	63.9 (46.0–81.5)	74.8 (54.6–91.0)	NA	NA	55.2 (38.3–73.7)	63.6 (43.6–83.1)	61.1 (41.6–80.8)	NA
Black or African American, NH	88.7 (85.7–91.2)	93.3 (90.3–95.6)^¶¶¶^	85.6 (79.7–90.5)^§§§^	NA	88.1 (84.9–90.9)	90.9 (86.9–94.1)	83.5 (76.2–89.6)	NA	79.4 (75.6–82.9)	84.6 (79.5–89.1)	NA	NA	63.1 (58.4–67.8)	66.1 (59.6–72.5)	59.4 (51.7–67.2)	NA
Hispanic or Latino	87.7 (84.4–90.6)	90.6 (87.7–93.1)	86.5 (79.9–91.9)	NA	88.6 (85.2–91.6)	88.2 (83.6–92.0)	82.6 (74.9–89.0)	NA	77.6 (73.5–81.5)	76.9 (71.9–81.6)	75.6 (68.7–81.9)	NA	61.0 (56.6–65.5)	61.0 (55.5–66.6)	56.4 (48.6–64.5)	NA
White, NH	90.7 (89.5–91.8)	90.9 (89.5–92.2)	87.9 (85.5–90.1)^§§§,¶¶¶^	NA	88.2 (86.7–89.6)	88.1 (86.5–89.7)	87.7 (84.5–90.6)	NA	71.4 (69.5–73.3)	72.7 (70.4–75.0)	70.3 (66.3–74.2)	NA	55.3 (53.2–57.4)	56.6 (54.0–59.2)	50.2 (46.9–53.6) ^§§§,¶¶¶^	NA
**Health insurance status**																
Private insurance only	90.2 (88.7–91.6)	92.0 (90.7–93.2)	88.0 (85.4–90.3)^§§§^	NA	89.7 (88.2–91.1)	89.4 (87.2–91.5)	88.9 (85.3–91.9)	NA	74.3 (72.3–76.3)	75.4 (72.6–78.1)	74.0 (69.8–78.1)	NA	59.1 (56.9–61.3)	60.4 (57.3–63.5)	54.6 (50.8–58.5)^§§§^	NA
Any Medicaid insurance	88.9 (86.7–90.8)	90.2 (87.9–92.2)	85.6 (81.5–89.2)^§§§^	NA	88.7 (86.4–90.8)	87.8 (85.2–90.0)	84.2 (79.7–88.2)	NA	77.7 (74.7–80.5)	77.8 (74.8–80.7)	72.7 (68.1–77.2)	NA	60.4 (57.1–63.7)	61.3 (57.6–65.1)	56.3 (51.1–61.7)	NA
Other insurance	89.2 (86.0–91.9)	89.8 (85.9–93.0)	95.1 (92.0–97.2)^§§§,¶¶¶^	NA	86.5 (82.5–90.0)	89.6 (85.9–92.7)	87.0 (76.7–94.3)	NA	72.5 (67.6–77.1)	77.9 (72.6–82.8)	68.9 (57.8–79.3)	NA	52.2 (46.3–58.3)	55.8 (49.2–62.6)	48.9 (38.6–60.3)	NA
Uninsured	80.7 (72.4–87.8)	84.0 (73.7–91.9)	82.3 (59.8–96.3)	NA	71.2 (61.1–80.6)	90.0 (80.2–96.3)^¶¶¶^	NA	NA	52.1 (41.6–63.4)	70.4 (57.2–82.6)^¶¶¶^	58.1 (39.3–78.0)	NA	38.4 (28.2–50.9)	37.1 (26.9–49.7)	28.3 (16.3–46.1)	NA

**Abbreviations:** AI/AN = American Indian or Alaska Native; HPV = human papillomavirus; MenACWY = quadrivalent meningococcal conjugate vaccine; MSA = metropolitan statistical area; NA= not applicable; NH = non-Hispanic; Tdap = tetanus, diphtheria, and acellular pertussis vaccine; UTD = up to date.

* Data for the 2006 birth year are from survey years 2019, 2020, 2021, and 2022; data for the 2007 birth year are from survey years 2020, 2021, and 2022; data for the 2008 birth year are from survey years 2021, and 2022; data for the 2009 birth year are from survey year 2022.

^†^ Includes vaccinations received by age 13 years (before the 13th birthday) and by age 14 years (before the 14th birthday).

^§^ MSA status was determined from household reported city and county of residence and was grouped into three categories: MSA principal city, MSA nonprincipal city, and non-MSA. MSA nonprincipal city and MSA principal city were as defined by the U.S. Census Bureau (https://www.census.gov/programs-surveys/metro-micro.html). Non-MSAs include urban populations not located within an MSA and completely rural areas.

^¶^ Adolescents were classified as being below the federal poverty level if their total family income was less than the level specified for the applicable family size and number of children and adolescents aged <18 years. All others were classified as at or above the federal poverty level (https://www.census.gov/data/tables/time-series/demo/income-poverty/historical-poverty-thresholds.html). Poverty status was unknown for 435 adolescents.

** Adolescents’ race and ethnicity was reported by their parent or guardian. Adolescents identified in this report as White, Black or African American, Asian, American Alaska Native or Indian, Native Hawaiian or other Pacific Islander, or multiple races were reported by the parent or guardian as non-Hispanic. Adolescents identified as having multiple races had more than one race category selected. Adolescents identified as Hispanic or Latino might be of any race. Estimates for Native Hawaiian or other Pacific Islander and multiracial adolescents were suppressed because of small sample size.

^††^ Adolescents’ health insurance status was reported by their parent or guardian. “Other insurance” includes the Children’s Health Insurance Program, military insurance, Indian Health Service, and any other type of health insurance not mentioned elsewhere.

^§§^ Estimates with 95% CIs >20 might not be reliable. Estimates with sample size <30 were suppressed and marked with NA.

^¶¶^ Statistically significant difference (p<0.05) in estimated vaccination coverage by age 13 years; referent group was 2007 birth year.

*** Statistically significant difference (p<0.05) in estimated vaccination coverage by age 13 years; referent group was 2008 birth year.

^†††^ Adolescents in the 2009 birth cohort reach their 14th birthday in 2023, and thus vaccinations by their 14th birthday in 2023 were not assessed by the 2022 NIS-Teen. These table cells were marked NA.

^§§§^ Statistically significant difference (p<0.05) in estimated vaccination coverage by age 14 years; referent group was 2007 birth year.

^¶¶¶^ Statistically significant difference (p<0.05) in estimated vaccination coverage by age 14 years; referent group was 2006 birth year.

By age 14 years, among adolescents born in 2008, coverage with ≥1 Tdap dose was 3–4 percentage points lower, and HPV UTD status was 5.0–6.0 percentage points lower among adolescents living at or above the federal poverty level,^†††††^ those who were non-Hispanic White, and those privately insured than among those born in 2007. Among adolescents born in 2008, coverage with ≥1 Tdap dose by age 14 years was 4.3 percentage points lower among those living in mostly suburban areas^§§§§§^ and 4.6 percentage points lower among those insured by Medicaid than among those born in 2007. All four vaccine measures ranged from 3.9 to 11.7 percentage points lower among those living in mostly urban areas in the 2008 birth cohort compared with the 2007 birth cohort.

## Discussion

This report used two analyses of 2022 NIS-Teen data to examine vaccination coverage among U.S. adolescents: birth cohort analyses were conducted to assess recent trends in vaccination coverage and a cross-sectional analysis evaluated coverage among adolescents aged 13–17 years during 2022. The birth cohort analysis identified lower coverage with ≥1 Tdap dose and ≥1 MenACWY dose by age 13 years, and lower coverage with ≥1 Tdap dose, ≥1 HPV dose, and HPV UTD by age 14 years, among adolescents born during 2008 (i.e., those who reached their 12th birthday during 2020) compared with those born during 2007. The continued lower coverage by age 14 years indicates that vaccination coverage did not rebound among this birth cohort in 2022. Coverage with all routinely recommended vaccines among adolescents born during 2008 and living in mostly urban areas was lower than coverage among those born during 2007, indicating that pandemic disruptions might have differentially affected urban areas. In contrast to findings for the 2008 birth cohort, coverage by age 13 years was not lower for the 2009 birth cohort compared with the two earlier birth cohorts, perhaps because these adolescents had an additional year after the peak of the pandemic to receive routinely recommended vaccines before becoming overdue, and because many primary care offices returned to normal operations.

The cross-sectional analysis showed that for the first time since 2013, HPV vaccination initiation did not increase among adolescents aged 13–17 years. HPV vaccination initiation fell among adolescents insured by Medicaid and remained lowest among the uninsured (two of the four groups that constitute the Vaccines for Children [VFC]–eligible population), highlighting the continued need for outreach among adolescents eligible for VFC.^¶¶¶¶¶^ VFC vaccine ordering data provide additional evidence that HPV vaccination coverage might be declining in VFC-eligible populations. VFC provider orders for HPV vaccines decreased 24% during 2020, 9% during 2021, and 12% during 2022 compared with 2019, and provider orders for non-HPV vaccines have rebounded to prepandemic levels (Whitlatch F, CDC unpublished data, 2023). The VFC program is vital to reach and administer vaccines to eligible adolescents to maintain vaccination coverage in underserved communities.

### Limitations

The findings in this report are subject to at least two limitations. First, selection bias due to low household response rate might have occurred if selected participants differed systematically from nonparticipants (*3*). Second, data were weighted to account for nonresponse and households without telephones, but some bias might remain. Recent total survey error assessments indicated that NIS-Teen estimates might underestimate actual coverage, with the largest underestimation occurring for Tdap (−5.0 percentage points) (*4**,**5*). In addition, the findings suggested no evidence of change in accuracy of NIS-Teen estimates from 2021 to 2022 for routine adolescent vaccines and for most catch-up vaccines (*5*).

### Implications for Public Health Practice

In the wake of the COVID-19 pandemic, many families might have missed well-child appointments when vaccinations were due (*6*). Ensuring that adolescents are up to date with recommended vaccines (Tdap, MenACWY, and HPV vaccine) is the best way to protect them from vaccine-preventable diseases. Particular focus is needed for subgroups that experienced larger recent declines in vaccination coverage or substantially lower coverage, including those born during 2008 and VFC-eligible populations. Resources for supporting catch-up vaccination are available at https://www.cdc.gov/vaccines/partners/routine-immunizations-lets-rise.html.

## Footnotes

* COVID-19 vaccination is recommended for all persons aged ≥6 months (https://www.cdc.gov/coronavirus/2019-ncov/vaccines/stay-up-to-date.html). Estimates of COVID-19 vaccination coverage are available at https://covid.cdc.gov/covid-data-tracker/#vaccination-states-jurisdictions and https://www.cdc.gov/vaccines/imz-managers/coverage/covidvaxview/interactive/children.html.

† Influenza vaccination is recommended for all persons aged ≥6 months. Influenza vaccination coverage estimates are available at https://www.cdc.gov/flu/fluvaxview/index.htm.

§ Meningococcal conjugate vaccination coverage represents coverage with the quadrivalent meningococcal conjugate vaccine or meningococcal-unknown type vaccine.

¶ Tetanus toxoid, reduced diphtheria toxoid, and acellular pertussis vaccine coverage represents coverage with ≥1 Tdap dose at age ≥10 years.

** Persons living in all identified households with mobile telephones were eligible for interview. Sampling weights were adjusted for survey nonresponse, adolescent multiplicity (number of chances of selection), and noncoverage of the survey sampling frame, and were calibrated to known population totals. During 2015–2017, NIS-Teen sampled from a landline frame in addition to a mobile telephone frame; therefore, sampling weights were also adjusted for overlapping samples of mixed telephone users. https://www.cdc.gov/vaccines/imz-managers/coverage/teenvaxview/pubs-presentations/dual-to-single-frame-teen.html

†† Local areas that received federal immunization funds under Section 317 of the Public Health Service Act were sampled separately. Those areas included Chicago, Illinois; New York, New York; Philadelphia County, Pennsylvania; Bexar County, Texas; and Houston, Texas. Two territories were sampled separately in 2022: Guam and Puerto Rico.

§§ The 2022 NIS-Teen sample included 7,623 females and 8,420 males. Adolescents from Guam (240) and Puerto Rico (671) were excluded from the national estimates.

¶¶ Estimates in this report include persons who might have received vaccinations on time or as catch-up. Influenza vaccination coverage data are not included in this report but are available at https://www.cdc.gov/flu/fluvaxview/index.htm.

*** The Council of American Survey Research Organizations response rate is the product of three other rates: 1) the resolution rate (the proportion of telephone numbers that can be identified as either business or residence), 2) the screening rate (the proportion of qualified households that complete the screening process), and 3) the cooperation rate (the proportion of contacted eligible households for which a completed interview is obtained).

††† Teens with at least one non–COVID-19 vaccination reported by a provider and those who had received no vaccinations were considered to have adequate provider data. “No vaccinations” indicates that the vaccination status is known because the parent or guardian indicated there were no vaccinations and the providers returned no immunization history forms or returned them indicating that no vaccinations had been administered.

§§§
https://www.cdc.gov/vaccines/imz-managers/nis/downloads/NIS-TEEN-PUF21-DUG.pdf

¶¶¶ 45 C.F.R. part 46.102(l)(2); 21 C.F.R. part 56; 42 U.S.C. Sect. 241(d); 5 U.S.C. Sect. 552a; 44 U.S.C. Sect. 3501 et seq.

**** Hepatitis A; hepatitis B; varicella; and measles, mumps, and rubella vaccines are considered childhood vaccinations and are recommended for adolescents who are not up to date with these vaccinations. Except as noted, coverage estimates for ≥1 and ≥2 varicella vaccine doses were obtained among adolescents with no history of varicella disease.

†††† MenB vaccination is not routinely recommended for all adolescents. Vaccines are administered to adolescents and young adults aged 16–23 years based on individual shared clinical decisionmaking. Coverage estimates for ≥1 and ≥2 MenB doses were calculated among adolescents who were aged 17 years at the time of interview.

§§§§ HPV vaccination coverage includes receipt of any HPV vaccine and does not distinguish between nine-valent (9vHPV), quadrivalent (4vHPV), or bivalent (2vHPV) vaccines. Some adolescents might have received more than the 2 or 3 recommended HPV vaccine doses.

¶¶¶¶ HPV up to date includes adolescents with ≥3 doses, and those with 2 doses when the first HPV vaccine dose was initiated at age <15 years and there was ≥5 months minus 4 days between the first and second dose (https://www.cdc.gov/vaccines/programs/iis/cdsi.html). This update to the HPV vaccination recommendation occurred in December 2016. Some adolescents might have received more than the 2 or 3 recommended HPV vaccine doses.

***** NIS-Teen data during 2015–2022 were combined, and Kaplan-Meier methods were used to calculate cumulative vaccination coverage estimates by age in days, stratified by annual birth cohort (2006 = 13,251; 2007 = 9,234; 2008 = 5,036; and 2009 = 1,655).

††††† Poverty status was unknown for 435 adolescents. Adolescents were classified as being below the federal poverty level if their total family income was less than the level specified for the applicable family size and number of children and adolescents aged <18 years. All others were classified as at or above the federal poverty level. https://www.census.gov/data/tables/time-series/demo/income-poverty/historical-poverty-thresholds.html

§§§§§ Metropopulation statistical area (MSA) status was determined from household reported city and county of residence and was grouped into three categories: MSA principal city, MSA nonprincipal city, and non-MSA. Non-MSAs include urban populations not located within an MSA and completely rural areas. https://www.census.gov/programs-surveys/metro-micro.html

¶¶¶¶¶ Persons aged ≤18 years who are Medicaid-eligible, uninsured, or American Indian or Alaska Native (as defined by the Indian Health Care Improvement Act) are eligible to receive vaccines from providers through the VFC program. Children categorized as underinsured because their health plans do not include coverage for recommended vaccinations are eligible to receive VFC vaccines if they are served by a rural health clinic or federally qualified health center or under an approved deputization agreement. https://www.cdc.gov/vaccines/programs/vfc/providers/eligibility.html
